# Heterogeneous mechanisms for synchronization of networks of resonant neurons under different E/I balance regimes

**DOI:** 10.3389/fnetp.2022.975951

**Published:** 2022-09-30

**Authors:** Jiaxing Wu, Sara J. Aton, Victoria Booth, Michal Zochowski

**Affiliations:** ^1^ Applied Physics Program, University of Michigan, Ann Arbor, MI, United States; ^2^ Department of Molecular, Cellular and Developmental Biology, University of Michigan, Ann Arbor, MI, United States; ^3^ Department of Mathematics, University of Michigan, Ann Arbor, MI, United States; ^4^ Department of Anesthesiology, University of Michigan Medical School, Ann Arbor, MI, United States; ^5^ Department of Physics, University of Michigan, Ann Arbor, MI, United States; ^6^ Biophysics Program, University of Michigan, Ann Arbor, MI, United States

**Keywords:** synchronization, neural oscillations, E/I balance, spatio-temporal network dynamics, computational modeling

## Abstract

Rhythmic synchronization of neuronal firing patterns is a widely present phenomenon in the brain—one that seems to be essential for many cognitive processes. A variety of mechanisms contribute to generation and synchronization of network oscillations, ranging from intrinsic cellular excitability to network mediated effects. However, it is unclear how these mechanisms interact together. Here, using computational modeling of excitatory-inhibitory neural networks, we show that different synchronization mechanisms dominate network dynamics at different levels of excitation and inhibition (i.e. E/I levels) as synaptic strength is systematically varied. Our results show that with low synaptic strength networks are sensitive to external oscillatory drive as a synchronizing mechanism—a hallmark of resonance. In contrast, in a strongly-connected regime, synchronization is driven by network effects via the direct interaction between excitation and inhibition, and spontaneous oscillations and cross-frequency coupling emerge. Unexpectedly, we find that while excitation dominates network synchrony at low excitatory coupling strengths, inhibition dominates at high excitatory coupling strengths. Together, our results provide novel insights into the oscillatory modulation of firing patterns in different excitation/inhibition regimes.

## Introduction

Synchronization of firing between neurons has been observed in a wide variety of brain processes and different brain regions (Diesmann et al., 1999; Steriade 2001; Brovelli et al., 2004; Buzsáki and Draguhn 2004; Lakatos, Shah et al., 2005, Buzsaki 2006, Kumar, Rotter et al., 2010; Fell and Axmacher 2011; Brunet et al., 2014; Babapoor-Farrokhran et al., 2017; Zhou et al., 2019). Coherent firing patterns are thought to be essential for multiple cognitive functions, including multi-modal information integration, memory consolidation, information transfer, and integration of information across large-scale, distributed neuronal organizations (Varela, Lachaux et al., 2001, Buzsaki 2006, Fell and Axmacher 2011; Buzsáki and Watson 2012; Breton et al., 2019). Abnormal neuronal synchronization is associated with cognitive dysfunction in epilepsy, schizophrenia, and other disorders (Spencer et al., 2003; Lewis et al., 2005; Uhlhaas and Singer 2006; Başar and Güntekin 2008; Lisman and Buzsáki 2008; Ferrarelli et al., 2010). Large scale oscillatory patterning - a direct consequence of neuronal synchronization - is thought to mediate information transfer throughout disparate brain regions (Fell and Axmacher 2011; Buzsáki and Watson 2012; Hahn et al., 2019; Gonzalez et al., 2020). While both synchrony and large scale oscillations are closely related, they can be distinct and can happen on different time-scales, thus able to mediate binding of different signal features for example (Hahn et al., 2019). Cross-frequency coupling of nested synchronous oscillatory dynamics supports the organization of information transfer in top-down and bottom-up functions (Axmacher et al., 2010; Fell and Axmacher 2011; Aru et al., 2015; Naze et al., 2018; Palva and Palva 2018).

At the same time, a large body of research has shown that excitatory/inhibitory (E/I) balance emerges within brain networks (Wehr and Zador 2003; Liu et al., 2009; Poo and Isaacson 2009; Stettler and Axel 2009; Tan et al., 2013; D'Amour J and Froemke 2015) and is important for regulating spatio-temporal activity patterns (van Vreeswijk and Sompolinsky 1996; Deneve and Machens 2016; Borges et al., 2020). E/I balance is observed in evoked responses to external stimuli, but is also present during spontaneous brain activity (Atallah and Scanziani 2009; Murphy and Miller 2009; Graupner and Reyes 2013). E/I balance can be loose (van Vreeswijk and Sompolinsky 1998; Brunel 2000; Salinas and Thier 2000; Rudolph et al., 2007), i.e. happening over long time scales, but recent findings have demonstrated that inhibition can closely track excitation at millisecond timescales, leaving a brief window of disinhibition for neurons to fire. This “tight balance” has been observed in different brain regions (Okun and Lampl 2008; Atallah and Scanziani 2009) and is now thought to play a significant role in learning and memory (Letzkus, Wolff et al., 2015) during various behavioral cycles (Niethard et al., 2016; Niethard et al., 2018). E/I balance can also regulate the occurrence of cortical up- and down-states (Shu et al., 2003; Haider et al., 2006).

The focus of this paper is to use computational modeling to investigate how synchrony and evolution of network-level excitation and inhibition mediate and interact with each other, affecting spatio-temporal patterning in the network, particularly large scale oscillatory activity.

While synchronous network oscillations have been widely characterized, there is still limited understanding regarding how structural network properties contribute to their emergence and interactions. In the hippocampus alone, different types of GABAergic interneurons are demonstrated to drive the emergence of synchrony via different signaling mechanisms (Hilscher et al., 2017) (Wester and McBain 2016). However, how the coordination of excitation and inhibition evolves due to changing network states or connectivity and contributes to generation of synchronous activity in the network cooperatively or alternatively still remains not fully understood.

In general, the mechanisms generating synchronous oscillations can be divided into two classes: 1) network mediated and 2) those driven by cell intrinsic properties—i.e., neuronal excitability. The prominent example of the first class is the so called pyramidal-interneuron gamma (PING) (Traub et al., 1996; Buzsáki and Wang 2012) mechanism and its derivatives. Here the oscillation emerges as a close interaction of excitation and inhibition in the network. Strong excitation triggers an inhibitory burst which feeds back onto the excitatory neurons, effectively shutting them down for a period of time. Lifting this inhibition leads to another burst of excitatory activity, thus repeating the process. In this case the inhibitory patterning is based on the feedback interactions of excitatory and inhibitory sub-networks rather than excitability properties of individual neurons. The second class depends on intrinsic membrane properties of individual neurons. These properties include resonant, subthreshold oscillations and/or so called type 2 excitability that promotes synchronization of neuronal spiking patterns (Prescott et al., 2008). These resonator-type neurons can be recruited effectively through an oscillating local field potential (LFP) and mediate coherent activity throughout distant brain regions as well as contribute to the interplay between brain rhythms of different frequency bands (Varela et al., 2001). Coherent, subthreshold membrane potential oscillations are thought to play an important role in functional selection and grouping (Engel et al., 2001). Computational modeling studies have highlighted how periodically-timed input to networks of neurons with and without resonant excitability strongly influences neural firing patterns (Lau and Zochowski 2011; Shtrahman and Zochowski 2015; Roach et al., 2018; Masoliver and Masoller 2020; Hansen et al., 2022).

The specific unresolved questions that remain are: to what degree can these two classes of synchronizing mechanisms coexist within the same network? What promotes dominance of one mechanism over another within a network? And how does emergence of synchronous oscillations temporally regulate the E/I balance within a network in the presence and absence of external oscillatory drive? Here we use biophysical, computational models of excitatory-inhibitory neural networks to demonstrate how external oscillatory drive interacts with intrinsic network dynamics to synchronize networks composed of neurons with type 2 excitability, at different global levels of excitation and inhibition. By systematically varying synaptic strengths, we demonstrate multiple dynamic regimes displaying heterogeneous network firing patterns and identify two distinct synchronization mechanisms emerging as a function of the interplay between excitation and inhibition. Our results show that when excitatory synaptic strength is relatively low, neural subthreshold membrane oscillations, coupled with external resonant driving current are able to generate ordered spiking, increase synchronization, and constrain the E/I ratio to a balanced state. On the other hand, when the network is strongly connected by excitatory synapses, global synchronization is generated by the interaction between synaptic excitation and inhibition. In addition, although external oscillatory drive modulates network dynamics, its effects vary depending on network properties, from generating detailed firing patterns within synchronous network bursts to modulating inter-burst intervals. Finally, we show that, counterintuitively, synchrony at low excitatory synaptic coupling is dominated by excitation while at high excitatory synaptic coupling, synchrony is dominated by inhibition.

## Methods

### Neuron model

**FIGURE 1:**
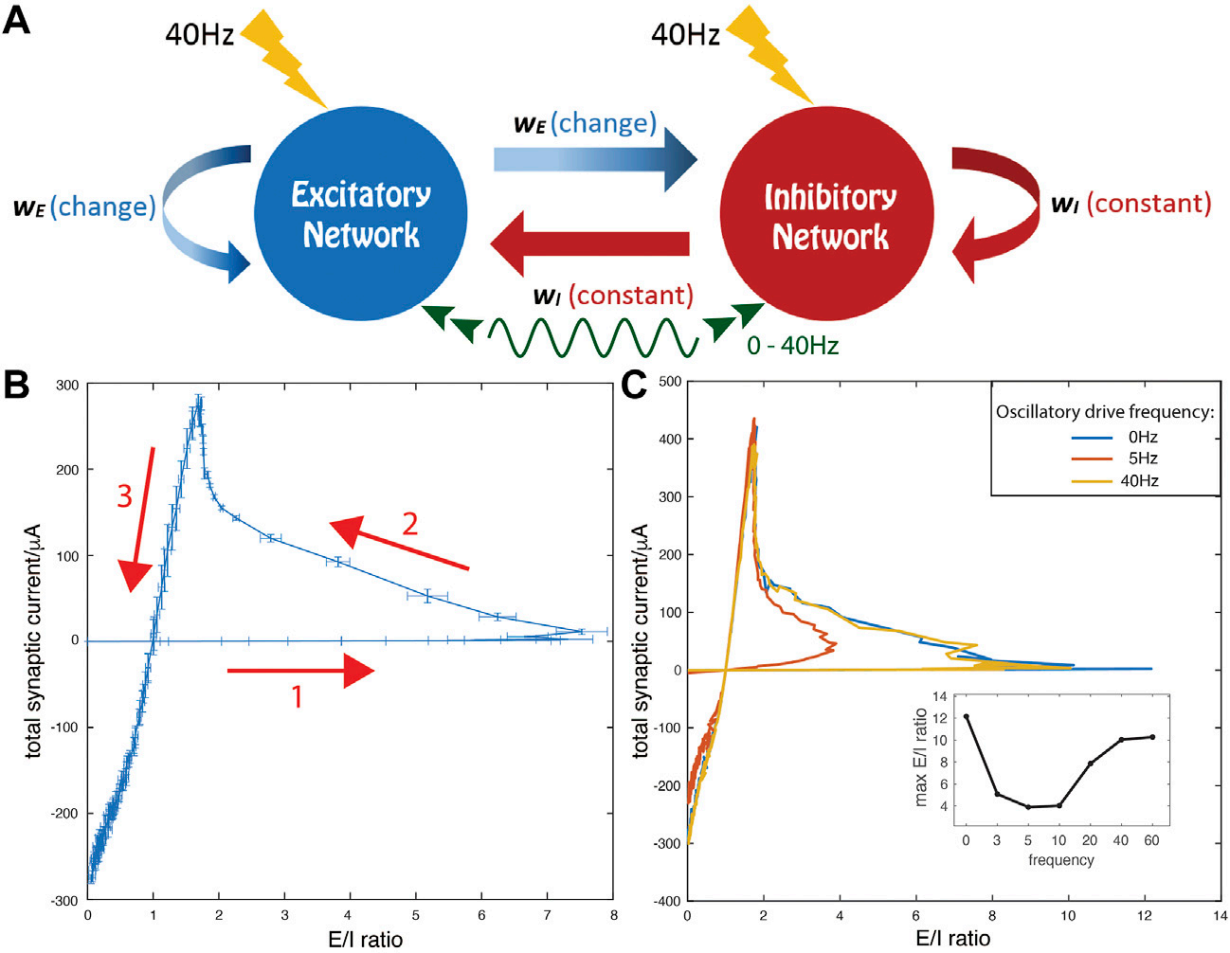
Model network and trajectories of measures of synaptic E and I currents for increasing excitatory synaptic strength, w_E_. **(A)** Schematic of model network consisting of excitatory and inhibitory cells randomly coupled with all excitatory (inhibitory) synaptic strengths set to w_E_ (w_I_). All cells receive oscillatory external drive currents at frequencies between 0 and 40 Hz (green curve) and random noisy current inputs (yellow lightning bolts). **(B,C)** Total synaptic current (E—I difference) is on the *y*-axis and E/I ratio is on the *x*-axis. The inhibitory synaptic strength w_I_ is fixed at 0.3 mS/cm^2^ while the excitatory synaptic strength, w_E_, is increased linearly from 0 to 2 mS/cm^2^. **(B)** No external oscillatory drive is present. The arrows mark the direction of the evolution of the E-I current measures as a function of increasing w_E_. Error bars indicate SE over 10 simulation runs with random initial conditions and different random network realizations. **(C)** Comparison of trajectories in the absence (0Hz, blue) and presence of external oscillatory drive at resonant frequency of 5 Hz (red) and non-resonant frequency of 40 Hz (yellow). (Inset) The maximum E/I ratio on each trajectory curve under external oscillatory stimulation at different frequencies. In this panel and in subsequent figures, results shown are averages over three simulation runs with random initial conditions and different random network realizations.

In the networks (schematic shown in Figure 1A), neurons are modeled using a modified Hodgkin-Huxley model with the addition of a slow, low-threshold K+ current. Due to this current, individual cells display type 2 phase response dynamics (Stiefel et al., 2008), with spike frequency adaptation and subthreshold, theta band membrane potential oscillations. Consequently the neurons act as resonators rather than integrators. The membrane voltage of each neuron is governed by:

$$C\frac{{dV}_{i}}{dt}=-g_{Na}m_{\infty }^{3}\left(V_{i}\right)h\left(V_{i}-V_{Na}\right)-g_{Kdr}n^{4}\left(V_{i}-V_{K}\right)-g_{L}\left(V_{i}-V_{L}\right)-g_{Ks}z\left(V_{i}-V_{K}\right)+I_{i}^{drive}+I_{i}^{noise}-I_{i}^{syn}$$ (1)

Each neuron receives a sub-threshold constant current input, which is sampled from a uniform distribution from $I_{i}^{DC}$ = [-0.8, 0.8] μA/cm^2^. In addition, the whole network is driven with a global sinusoidal current with amplitude A = 0.3 μA/cm^2^ (if not stated otherwise). Thus, the external drive is defined as:

$$I_{i}^{drive}=I_{i}^{DC}+A\sin\left(\omega t\right)$$ (2)

where ω is varied to yield oscillations of 0–60 Hz. Values of $I_{i}^{DC}$ and A are chosen so that all neurons display only sub-threshold membrane oscillations even at the peak of each sinusoidal cycle. Each neuron additionally receives Poisson random noisy current input $I_{i}^{noise}$ consisting of brief (0.05 ms), square, 30 μA/cm^2^ current pulses, delivered at average frequency of 40 Hz.

Ionic currents are gated as follows. For Na + channels:

$$m_{\infty }\left(V\right)={\left\{1+\exp\left[\frac{-V-30.0}{9.5}\right]\right\}}^{-1}$$ (3)

$$\frac{dh}{dt}=\frac{h_{\infty }\left(V\right)-h}{\tau_{h}\left(V\right)}$$ (4)

where $h_{\infty }\left(V\right)={\left\{1+\exp\left[\frac{V+53.0}{7.0}\right]\right\}}^{-1}$ , and $\tau_{h}\left(V\right)=0.37+2.78{\left\{1+\exp\left[\frac{V+40.5}{6.0}\right]\right\}}^{-1}$ .

The kinetics of the K+ delayed rectifier current are governed by:

$$\frac{dn}{dt}=\frac{n_{\infty }\left(V\right)-n}{\tau_{n}\left(V\right)}$$ (5)

with $n_{\infty }\left(V\right)={\left\{1+\exp\left[\frac{-V-30.0}{10.0}\right]\right\}}^{-1}$ , and $\tau_{n}\left(V\right)=0.37+1.85{\left\{1+\exp\left[\frac{V+27.0}{15.0}\right]\right\}}^{-1}$ .

The gating of the slow, low-threshold K+ current evolves as:

$$\frac{dz}{dt}=\frac{z_{\infty }\left(V\right)-z}{75.0}$$ (6)

with $z_{\infty }\left(V\right)={\left\{1+\exp\left[\frac{-V-39.0}{5.0}\right]\right\}}^{-1}$ .

The leak conductance is given by g_L_ = 0.02 mS/cm^2^. Other parameters are set to C = 1 μF/cm^2^, g_Na_ = 24.0 mS/cm^2^, g_Kdr_ = 3.0 mS/cm^2^, V_Na_ = 55.0mV, V_K_ = -90.0mV, and V_L_ = -60.0 mV.

### Network structure and dynamics

Networks were composed of 250 excitatory model neurons and 250 inhibitory model neurons connected randomly to each other based on a directed Erdos-Renyi-Gilbert random graph (Figure 1A). Connectivity density was 3%, providing approximately 15 out-going synaptic connections per cell. Although the excitatory to inhibitory cell ratio may not be physiological compared to some brain areas, we chose to keep the symmetry between the excitatory and inhibitory populations so that excitatory and inhibitory signaling could be directly compared and controlled by their relative synaptic strengths (see Discussion for more explanation).

The general form of the synaptic current transmitted from neuron j to neuron i is modeled as:

$$I_{ij}^{syn}=w\;\exp\left(-\frac{t-t_{j}}{\tau }\right)\left(V_{i}-E_{syn}\right)$$ (7)

The parameter w represents the strength of synapses originating from excitatory cells (w_E_) or originating from inhibitory cells (w_I_). Specifically, all out-going synapses from excitatory cells have strength w_E_ regardless of their postsynaptic target (either another excitatory cell or an inhibitory cell). Likewise, w_I_ is the strength of all out-going synapses from inhibitory cells. The values of w_E_ and w_I_ will be changed systematically. t_j_ refers to the spiking time of neuron j, and τ is the synaptic constant time with value of 0.5 ms, simulating fast AMPA-like excitatory synapses or fast GABA-A-like inhibitory synapses. The reversal potential $E_{syn}$ is set to 0 mV for excitatory synaptic current and -75 mV for inhibitory current. Eventually the total synaptic input into a postsynaptic neuron is the summation of currents from the set of all connected presynaptic neurons $\Gamma_{i}$ , $I_{i}^{syn}=\sum_{j\in \Gamma_{i}}I_{ij}^{syn}$ .

The dynamics of the network were simulated from random initial conditions with a time step of 0.05 ms, using a fourth-order Runge-Kutta method. Dynamics were allowed to evolve to an asymptotically stable state, then network measures were computed over a 3 s time window. The results shown are averaged over three simulations, each from different random initial conditions and with different instantiations of the random network connectivity. To demonstrate the robustness of simulation results, Figure 1B shows average results for 10 simulations with random initial conditions and different random network realizations.

### Synchrony measurement

The measure of synchronization of the firing pattern in the network is adopted from (Golomb and Rinzel 1994) and is calculated as the averaged fluctuation over all the neurons normalized by the fluctuations of each neuron:

$$S_{N}=\sqrt{\frac{\sigma_{V}^{2}}{\frac{1}{N}\sum_{i=1}^{N}\sigma_{V_{i}}^{2}}}$$ (8)

To compute synchrony $S_{N}$ , each spike train is convolved with a Gaussian with a width of about 1/10 of the mean inter-spike interval to create a neuronal voltage trace. The specific choice of the width does not affect the results. Then $\sigma_{V}^{2}$ is calculated as the variance of the averaged neuronal voltage traces, while $\sigma_{V_{i}}^{2}$ indicates the variance of the voltage trace for the *i*th neuron. The values of $S_{N}$ lie between 0 and 1 for random firing and perfect synchrony, respectively.

### Quantification of input dependent spike recruitment

To quantify spike timing patterns within network bursts, the timestamps of spikes within each burst are extracted and differences with the timing of the first spike in the burst are computed. Neurons with higher driving constant current, $I_{i}^{DC}$ , are expected to fire earlier relatively in the burst (Roach et al., 2018; Roach et al., 2019), and the relationship between $I_{i}^{DC}$ and spike time within a burst turns out to be linear approximately. Next, a straight line is fitted using least-squares regression for this relationship. Regression line slopes are used as an index for cell recruitment ordering within bursts with a negative value representing the general temporal difference when neurons having various $I_{i}^{DC}$ values are recruited; slope of 0 means that all neurons fire synchronously in the burst with no time lags. The values of the slopes are averaged over all the bursts over the whole simulation time period.

## Results

### Total synaptic current and E/I ratio under different external oscillatory drives

In our model networks, we investigated how changes in relative coupling strength between excitatory and inhibitory sub-networks, in the presence or absence of external oscillatory drive, affect generation of synchronous network oscillations. We also tested how these activity states affect E/I balance in the network. To systematically study different regimes of connectivity strength, we set inhibitory synaptic weights (w_I_) constant at 0.3 mS/cm^2^ while varying excitatory weights (w_E_) from 0 to 5 × w_I_. We define total excitatory (E) current (arriving during time-period T) as the sum of all excitatory postsynaptic potentials (EPSPs, as defined by Eq. (7)) arriving at excitatory and inhibitory neurons, and conversely total inhibitory (I) current as the sum of all inhibitory postsynaptic potentials (IPSPs) arriving at both cell populations. We subsequently calculated the E/I current ratio of these two values as

$$\frac{E}{I}=\frac{\int_{0}^{T}\underset{i}{\sum }\sum_{j}\sum_{k}w_{E}\exp\left[\frac{t_{j,k}-t}{\tau }\right]\left(V_{i}\left(t\right)-E_{syn}^{E}\right)dt}{\int_{0}^{T}\underset{i}{\sum }\sum_{j}\sum_{k}w_{I}\exp\left[\frac{t_{j,k}-t}{\tau }\right]\left(V_{i}\left(t\right)-E_{syn}^{I}\right)dt}$$ (9)

where k denotes the spike number occurring in the *j*th pre-synaptic cell, j sums over all E cells in the numerator and over all I cells in the denominator, and i sums over all cells in the network. The difference E—I of these values, referred to as total synaptic current, was computed similarly.

We observed that the relationship between the E/I current ratio (*X*-axis) and the difference between E and I currents (*Y*-axis), with a linear increase of w_E_, forms a non-monotonic loop (Figure 1B). Initially, for weak but increasing w_E_ values, E/I ratio increases while the total current difference grows at a much slower rate (arrow 1). As w_E_ increases through intermediate values, E/I decreases while the current difference increases at a higher rate (arrow 2). Finally, as w_E_ increases through high values, both E/I ratio and the current difference rapidly decrease (arrow 3). Further, the detailed shape of the loop depends on the presence of external oscillatory drive and its frequency (Figure 1C). For weak excitatory coupling (w_E_), if the driving frequency is between 5 and 10 Hz (which matches the natural frequency of subthreshold membrane oscillations in the neuronal model), we observe a sharp decline in the maximal E/I ratio observed (red curve). However, if the oscillatory drive has a frequency outside this range, the loop’s shape (yellow curve) converges to that observed when no drive is present (blue curve). Interestingly, the part of the loop corresponding to strong excitatory coupling (i.e. high w_E_ values) does not change with frequency of external oscillatory drive. Thus, for weak w_E_, the network dynamical state quantified by E-I balance is highly dependent on the frequency of oscillatory drive, while for higher w_E_, network E-I dynamics are independent of oscillatory drive. Qualitatively similar results are obtained when the inhibitory synaptic strength w_I_ is larger, as shown in the (Supplementary Figure S1). However, increased inhibitory synaptic strength limits the maximum E/I ratio and thus the domain of the E/I trajectory loop, which is further diminished if the resonant oscillatory drive is present.

These results suggest that cellular resonance with oscillatory drive plays an important role in network dynamics when excitatory synaptic strength w_E_ is weak, while network dynamics are less influenced by the oscillatory drive at high w_E_. Therefore, we divide the network dynamical state into two regimes: 1) the resonance regime around the first turning point of the total current-vs-E/I trajectory curve (between arrows 1 and 2), and, 2) the network-driven regime after the second turning point of the trajectory curve (between arrows 2 and 3). Below, we will elucidate the properties of the dynamics and the resulting mechanisms for network synchronization in these two regimes.

### Cellular resonance in the weak excitatory coupling regime

**FIGURE 2:**
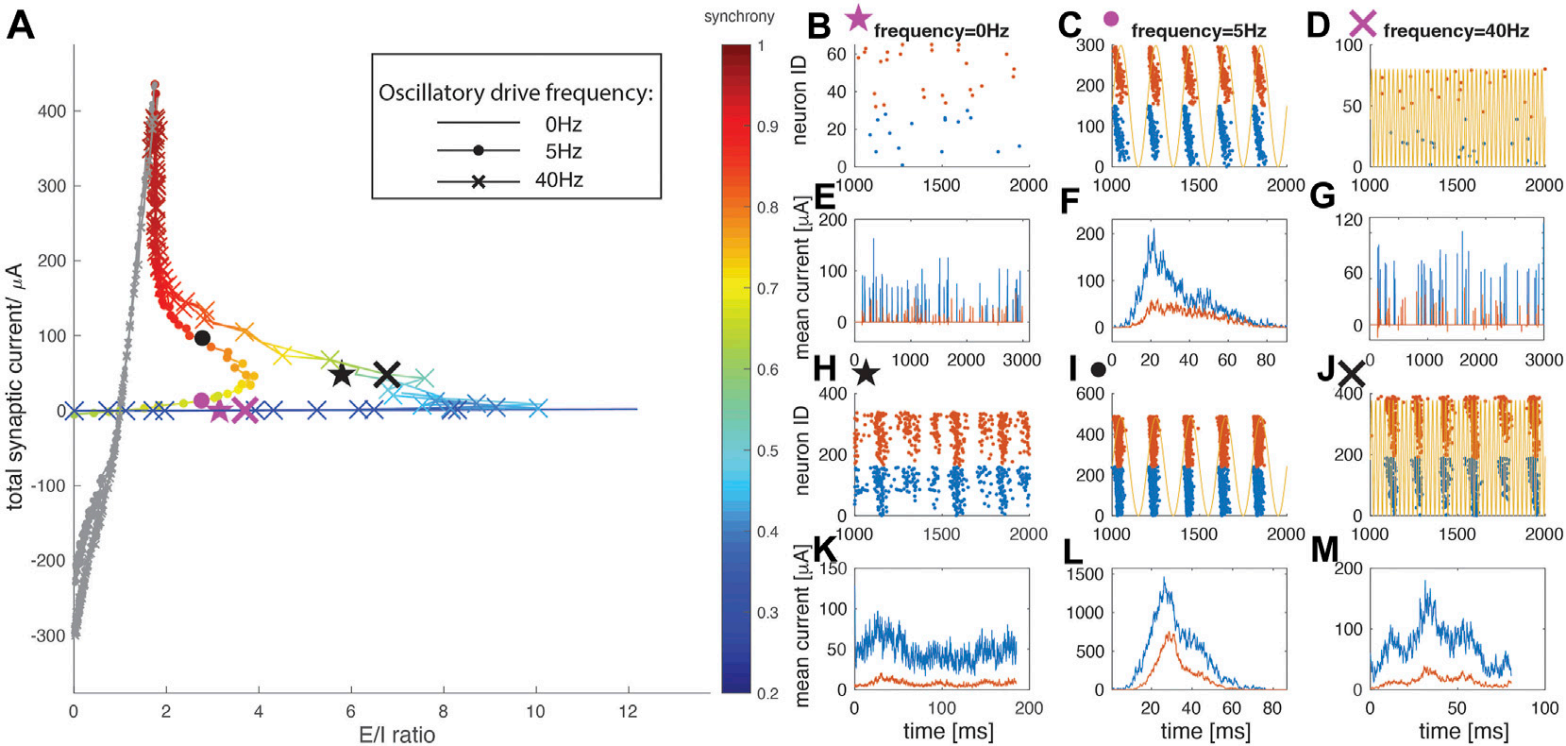
Network dynamics in the resonance regime (colored). **(A)** E/I ratio trajectories as w_E_ is increased under no oscillatory drive (plain curve), oscillatory drive at neuron natural frequency of 5 Hz (dot marker), and oscillatory drive at 40 Hz (cross marker). The color indicates the network synchrony measure. **(B–M)** Representative raster plots and burst-triggered averaged current traces for the data points labeled on the curves in panel **(A)**. Blue represents excitatory cells/currents, while red represents the inhibitory ones. Two representative data points are chosen for each driving condition, with a weaker w_E_ = 0.08 mS/cm^2^ and a stronger w_E_ = 0.24 mS/cm^2^ **(B,E)** No oscillatory driving with weaker w_E_ (pink star marker) **(C,F)** Oscillatory driving at resonant 5 Hz frequency with weaker w_E_ (pink dot marker) **(D,G)** Oscillatory driving at 40 Hz with weaker w_E_ (pink cross marker) **(H,K)** No oscillatory driving with stronger w_E_ (black star marker) **(I,L)** Oscillatory driving at 5 Hz with stronger w_E_ (black dot marker) **(J,M)** Oscillatory driving at 40 Hz with stronger w_E_ (black cross marker). Note that **(E)** and **(G)** are direct (i.e. not burst triggered) plots of the excitatory/inhibitory currents as there is no bursting activity. Yellow curves in **(C)**, **(D)**, **(I)** and **(J)** represent the external oscillatory driving current at 5 Hz in **(C)** and **(I)**, and at 40 Hz in **(D)** and **(J)**.

We first investigated the dynamical E/I ratio and spatio-temporal activity patterns for weak w_E_ (Figure 2A), at different frequencies of external drive. We measured the level of synchrony in the network, and depicted it as a color of the curve. The frequency of the driving oscillation was varied between 0 Hz (no oscillation) and 40 Hz. At these two boundary values, the horizontal extent of the E/I trajectory loop is the largest (i.e. maximal E/I ratio is achieved) and synchrony begins to appear as the trajectory approaches the second turning point (at highest w_E_ values in this regime). On the other hand, when the driving signal is a 5 Hz oscillation the network fires at an intermediate synchrony level (∼0.6) and the horizontal extent of the loop (i.e. the maximal E/I ratio, see inset Figure 1C) is significantly reduced. Given this strong frequency-dependence of the total current-vs-E/I trajectory loop, we identify this region as the resonance regime. We next investigated network-wide pattern formation as a function of the parametric position on the loop. To visualize the spatio-temporal patterning within the network, we display raster plots and calculate the burst triggered averages of excitatory and inhibitory currents in the network, for two w_E_ values, and for three oscillatory drive frequencies (0 Hz, 5 Hz, 40 Hz; markers in Figure 1A). When w_E_ is weak, (w_E_ = 0.08 mS/cm^2^; pink markers in Figure 2A, panels b, c, d depict raster plots and panels e, f, g depict the current traces, with blue indicating excitatory and red indicating inhibitory cells/traces respectively, and yellow curves represent the oscillatory driving current), spiking with 0 Hz (no oscillatory drive) and 40 Hz is sparse and random. In contrast, the 5 Hz oscillatory drive increases the firing rates of the neurons and network synchrony by regulating phase locking of spikes at a specific phase of the oscillation. Moreover, in this regime, the phase of locking systematically varies from cell to cell and depends on the constant current, $I_{i}^{DC}$ , that is applied to the cell (see Methods). To better depict this, we adjusted the *y*-axes of the raster plots so that cell order is a monotonic function of the cells’ constant current input, $I_{i}^{DC}$ . This ordering provides information about the relative magnitude of individual cell input and may underlie network structural reorganization if spike timing dependent plasticity is present. The analysis of burst triggered current averages (Figures 2E–G) reveal that within this regime, the dynamics is driven by resonant activation of excitatory cells, with inhibitory neuron activation being significantly weaker.

Moving along the trajectory loop to the region with higher excitatory weight (w_E_ = 0.24 mS/cm^2^, black markers in Figure 2A, panels h–j for raster plots and panels k–m for burst triggered current averages), the resonance mechanism for synchrony takes hold, and a spontaneous bursting pattern starts to emerge for no oscillatory drive (Figure 2G) and 40 Hz oscillatory drive (Figure 2I) conditions. In this case however, w_E_ is still relatively weak, and the burst triggered current traces indicate that inhibitory current contribution to network dynamics is still smaller than the contribution of excitatory current. This indicates that synchrony emerges via intrinsic cell mechanisms rather than interacting excitation and inhibition (i.e. a PING-like effect). With 5 Hz oscillatory drive, spiking patterns of individual cells remain phase-locked to the oscillation, and network dynamics are still dominated by excitatory activity. These spiking patterns did not change qualitatively for different values of inhibitory synaptic strengths (see Supplementary Figures S2 and S3 in supplemental data).

### Highly synchronized dynamics generated by interaction of network excitation and inhibition mechanisms

**FIGURE 3:**
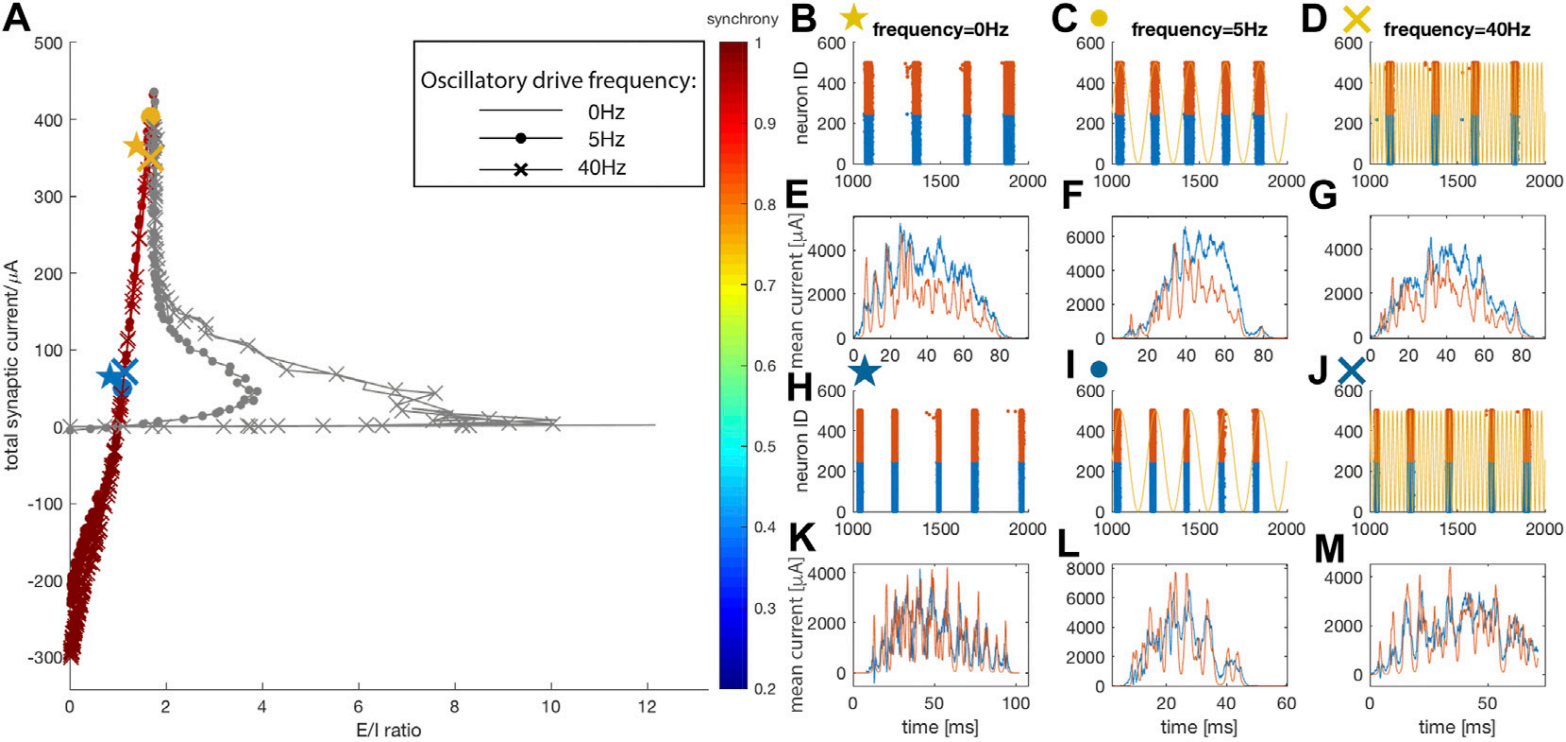
Network dynamics in the regime driven by PING-like mechanisms (colored). The panels correspond to those on Figure 2. **(A)** E/I ratio vs. total synaptic current trajectories. Similarly, two representative data points are chosen for each oscillatory driving condition, with a weaker (w_E_ = 0.66 mS/cm^2^) and a stronger (w_E_ = 0.84 mS/cm^2^) excitatory synaptic strength. Color reports level of synchronization. **(B–M)** Raster plots and burst triggered, averaged current traces for: **(B,E)** No oscillatory driving with weaker w_E_ (yellow star marker) **(C,F)** Oscillatory driving at 5 Hz with weaker w_E_ (yellow dot marker) **(D,G)** Oscillatory driving at 40 Hz with weaker w_E_ (yellow cross marker) **(H,K)** No oscillatory driving with stronger w_E_ (blue star marker) **(I,L)** Oscillatory driving at 5 Hz with stronger w_E_ (blue dot marker) **(J,M)** Oscillatory driving at 40 Hz with stronger w_E_ (blue cross marker). Yellow curves in **(C)**, **(D)**, **(I)** and **(J)** represent the external oscillatory driving current at 5 Hz in **(C)** and **(I)**, and at 40 Hz in **(D)** and **(J)**.

With increasing w_E_, the network enters the second regime in which network-driven synchrony is mediated via strong bursts of inhibition. Here the role of the external oscillatory drive in affecting E/I ratio diminishes, and the total current-vs-E/I trajectory curves for different oscillatory drive frequencies merge around the second turning point (Figure 3A). Network dynamics are likewise similar with different drive frequencies except for the phase locking of spiking activity with 5 Hz oscillatory drive. This indicates that at high w_E_ values, resonance effects of individual neurons’ firing with oscillatory input become less important, while intra-network interactions dominate the dynamics. While the degree of synchrony remains high in this regime, the network-wide firing pattern changes. Representative raster plots are shown on Figures 3B–D, and corresponding burst triggered average current traces on Figures 3E–G. When excitatory weight is w_E_ = 0.66 mS/cm^2^ (yellow markers), the system is around the second turning point and the magnitude of inhibition during network oscillatory firing starts to approach that of the excitation. While network excitation allows persistence of wide, multi-spike bursts, one can observe the emergence of a tight correlation between inhibitory and excitatory currents. For the highest values of excitatory coupling, (w_E_ = 0.84 mS/cm^2^, blue markers, and higher), the network moves into an inhibition-dominant regime. Here, not only is the firing pattern highly synchronous (Figures 3H–J), but each burst is composed of multiple single bursts. This interaction between excitatory and inhibitory currents, demonstrated in Figures 3K–M, explains the mechanism underlying the synchronous dynamics. At the beginning of each burst, excitatory current increases first. This burst of excitation leads to a strong burst of inhibition (at the peaks, the inhibitory current typically has a higher magnitude than excitatory current) which momentarily hyperpolarizes excitatory neurons. As the excitatory neurons recover, another excitatory burst is generated which is again followed by inhibitory neuron activation. Thus, here the synchrony is mediated by inhibition bursts, i. e, a PING-like mechanism (Traub et al., 1996; Buzsáki and Wang 2012), rather than by individual neurons’ resonance. For different values of inhibitory synaptic strength, the patterns remain highly synchronous (see Supplementary Figures S2 and S3 in supplemental data). However, the length of bursting periods increases with higher inhibition levels. This is due to the fact that inhibitory coupling also affects inhibitory neurons’ firing, reducing the inhibition needed to terminate the bursts.

### Modulation of the temporal relationship between excitatory and inhibitory currents under oscillatory drive

**FIGURE 4:**
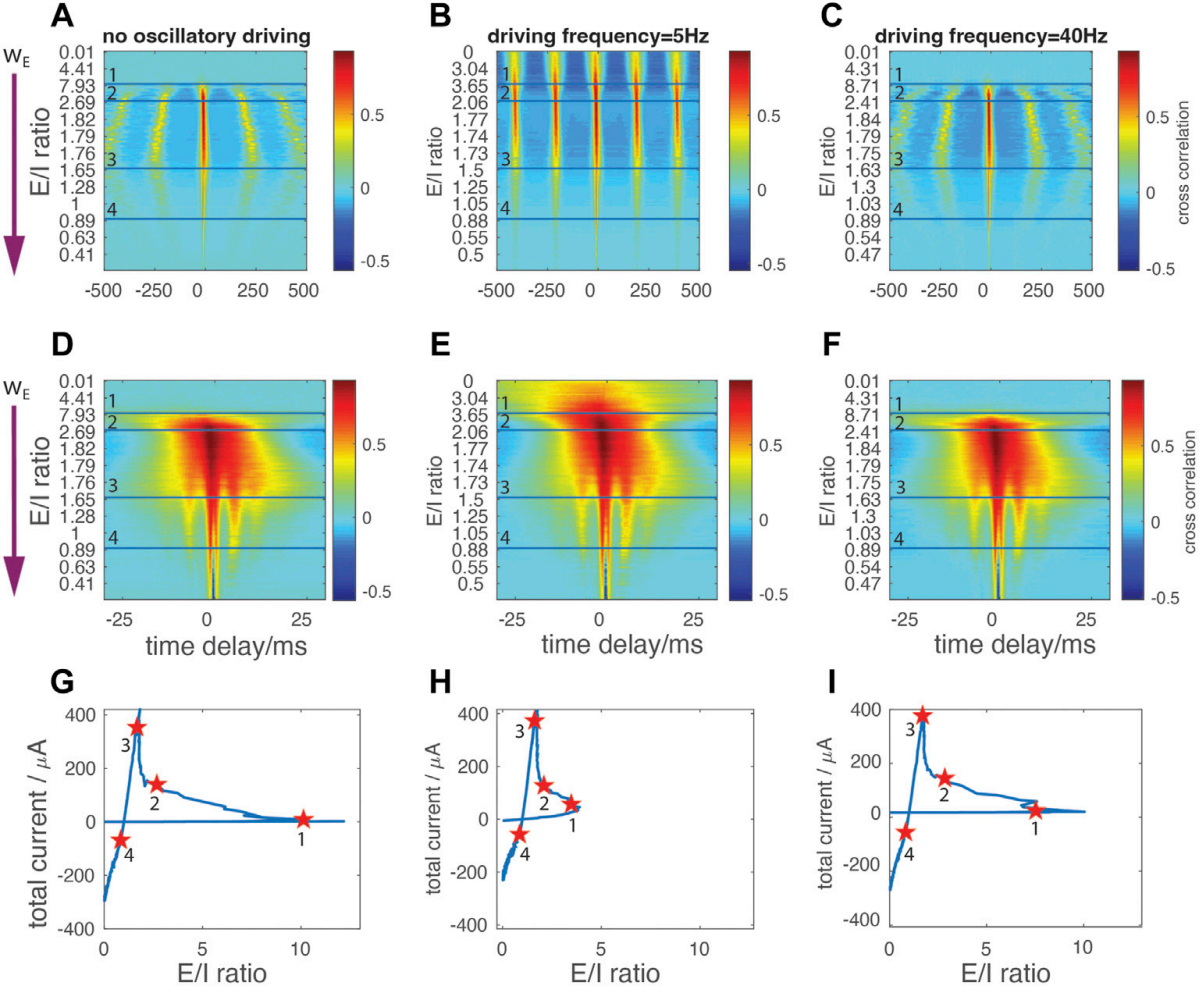
Temporal interdependencies between the excitatory and inhibitory currents **(A,B,C)** Cross correlation of network’s E and I currents for: no oscillatory drive **(A)**, 5 Hz (resonant) **(B)**, and 40 Hz **(C)** external oscillatory drive within time windows of 1s width **(D–F)** Cross correlation of network’s E and I currents for: no oscillatory drive **(D)**, 5 Hz **(E)**, 40 Hz driving **(F)** within time window of 60 ms width. *X*-axis denotes the time delay between the two currents; negative values denote excitation leading inhibition. Excitatory synaptic strength w_E_ increases from top to bottom (arrow in **(A)** and **(D)**), with the E/I ratio indicated on *Y*-axis. Horizontal lines correspond to star markers on the E/I vs total current curves in **(G–I)**, respectively.

We next focused on the changing temporal relationship between the excitatory and inhibitory currents as w_E_ increases (Figure 4), in the presence and absence of oscillatory drive. We calculated the cross-correlation between E and I currents within a 1-s window (Figures 4A–C), then examined tight temporal locking within a 60-ms window (Figures 4D–F). The *y*-axes of the correlation maps depict changes in w_E_. From top to bottom of these maps, w_E_ monotonically increases, resulting in the different E/I ratios within the network (*y*-axis tick labels). The horizontal lines on the maps mark the corresponding locations on the E/I trajectory curve, marked with star markers on Figures 4G–I moving counter-clock wise. We note that Figures 4A–C also give information on the frequency of network bursting activity, since the correlation peaks occur during network bursts and the harmonics in the correlation occur at multiples of the interburst interval.

For the weakest w_E_ coupling strengths (i.e., above the first horizontal separation line, corresponding to the initial part of the E/I ratio trajectory), no correlation patterns between excitatory and inhibitory currents are observed either when no external oscillation is present (Figures 4A–D) or with 40 Hz oscillatory input (Figures 4C,F). The network instead exhibits sparse random spiking. With 5 Hz input (Figures 4B,E), network burst activity is locked to the external oscillation and periodic temporal correlations emerge representing interburst intervals. As before, the external currents applied to each neuron are subthreshold indicating that, in this regime, the resonant drive effectively recruits neuronal firing at each cycle of the sinusoidal wave. The correlation peak is broad due to the temporal shift of firing pattern of the cells having different DC input, as observed on Figure 2C.

When w_E_ is increased, the excitation is strong enough to activate inhibition, thus decreasing the E/I ratio and causing the E/I trajectory to pass through the first turning point (Figures 4G–I). Between the first and second horizonal line (Figures 4A–F), corresponding to the first and second marker (Figures 4G–I), periodic correlation patterns spontaneously appear without external driving input (Figures 4A,D). In this regime, we observe a dynamic interaction between resonance and spontaneous oscillatory bursting. When the network is driven by an oscillation at resonant frequency, the inter-burst intervals are stabilized at 200 ms - corresponding to the period of 5 Hz oscillation (Figure 4B). In the non-resonant regime (i.e., no oscillatory drive or 40 Hz drive) inter-burst intervals change their duration monotonically with the increase of w_E_ (Figures 4A,C). Furthermore, with 5 Hz stimulation the correlation between two oscillatory cycles is higher, indicating more robust and systematic network activation (compare Figures 4A–C). These two features indicate that the external oscillatory drive at natural neuronal firing frequency is able to stabilize the firing pattern independently of changes of synaptic strength.

With further increases of w_E_ (to the range of values corresponding to the regime around the third marker), the system enters a network-driven PING-like regime and the trajectory undergoes the second turning point (Figures 4G–I). Here, while we continue to observe stabilization of inter-burst frequency when the resonant drive is present, the detailed temporal intra-burst pattern emerges with a cross-frequency phase coupling between the theta band (about 5 Hz) and fast gamma band (about 160 Hz) (illustrated by the emergence of fingering just above horizontal line three in Figures 4D–F). This indicates a large degree of synchrony within and between the excitatory and inhibitory populations. These network dynamics are now independent of external oscillatory drive.

### Resonance effects of oscillatory driving at various balance states

**FIGURE 5:**
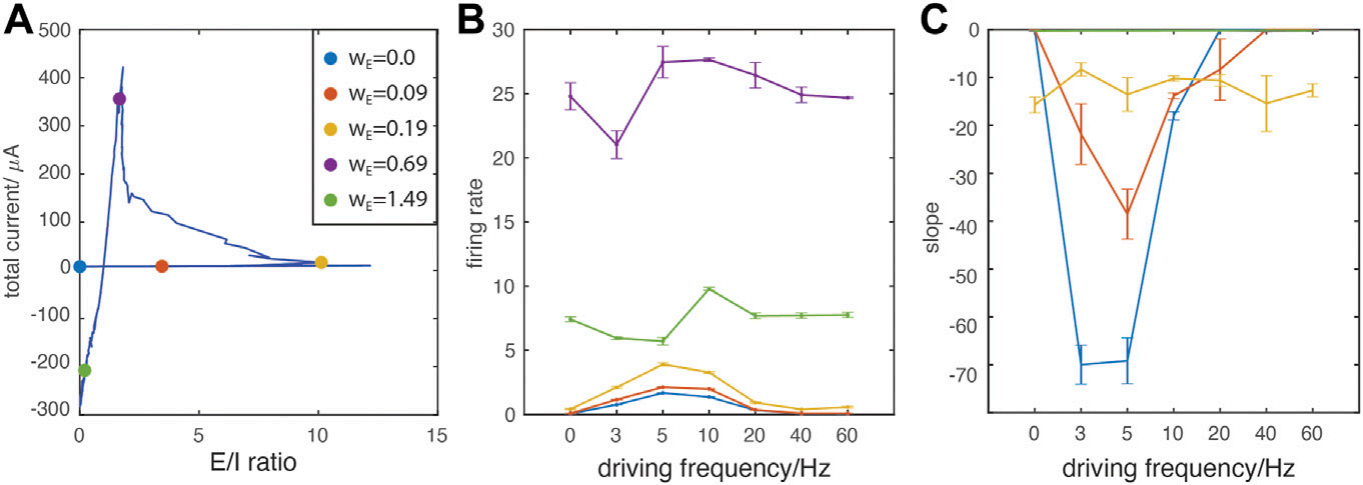
Oscillatory drive frequency dependent effects on network dynamics. **(A)** E/I ratio vs. total current trajectory indicating the location of representative data points on the curve (color dots). For every point the value of w_E_ increases in the following order: blue (lowest), red, yellow, violet, green (highest); **(B)** The network-wide firing rate as a function of oscillatory driving frequencies. **(C)** The index for cell recruitment ordering within synchronous bursts, as a function of magnitude of external constant drive, I^DC^ (see Methods). Note that for w_E_ = 0–0.09 mS/cm^2^ at 40 and 60 Hz no bursting activity occurs.

Finally, to characterize E/I mediated spatio-temporal patterning between the resonant regime and PING-like synchronous regime, we quantified dynamic network properties as a function of driving frequencies. We choose five representative w_E_ values at which we quantify the firing patterns (Figure 5A). These points correspond to: 1) no excitatory connections (blue), 2) a weak w_E_ when the oscillatory drive has significant impact on network firing (red), 3) around the first turning point where inhibition gets activated (resonance regime, yellow), 4) around the second turning point where the system transitions from resonance to a PING-like regime (violet), and finally, 6) the network is strongly connected (high w_E_, green). Figures 5B,C depicts changes in the network firing rate and the degree that spike time ordering within synchronous bursts is a function of constant input $I_{i}^{DC}$ applied to a neuron (see Methods). We observe that for weak coupling regimes (i.e. small w_E_), when the network is driven by the oscillations around the natural frequency (3–10 Hz) it displays not only an increased firing rate (Figure 5B) but also highly ordered neuronal recruitment within bursts, based on the intrinsic cell excitability (i.e. $I_{i}^{DC}$ magnitude), (Figure 5C). This input-dependent recruitment could possibly indicate the emergence of a temporal code that carries network-wide information about the relative magnitude of neuronal excitation. Such temporal coding may subsequently drive structural network reorganization if spike-timing dependent plasticity (STDP) is present. For non-resonant frequencies (40 Hz, 60 Hz) we find that this effect is absent.

The oscillation-ordered spiking is particularly prominent at the weakest w_E_ (blue line), indicating that this effect is gradually weakened by stronger network E/I interactions. Around the first turning point (yellow), firing rates also display noticeable resonance effects (Figure 5B), but the spike ordering is diminished significantly (Figure 5C). For stronger magnitude of w_E_, when PING-like bursting regime emerges (violet and green), changing frequencies of external oscillatory drive do not affect intra-burst spike dynamics (Figures 5B,C). The much higher firing rate at w_E_ around the second turning point (Figure 5B violet) results from wide bursts which contain random firing, in comparison to the lower firing rate of single spike bursting (Figure 5B green).

## Discussion

Here we use a biophysical model network of recurrently connected excitatory and inhibitory neurons with type 2 excitability to investigate co-dependence of different network synchrony mechanisms and E/I balance in the network. By systematically varying synaptic strength, the network E/I level forms a non-monotonic trajectory in the total current-vs-E/I ratio relationship. In previous work, we have shown that similar loop trajectories in the total current-vs-E/I ratio as excitatory synaptic strength is increased occur in networks consisting of model neurons with type 1 (i.e., integrator) excitability (Wu et al., 2020). In those networks, the qualitative shape of the trajectory loop did not depend on the ratio of E and I cells, the network connectivity density or the network connectivity topology. The present results further show this non-monotonic pattern of E/I regulation does not depend on cellular excitability.

In networks of cells with type 2 excitability, and thus resonant firing responses, for weak excitatory coupling, the E/I trajectory loop was strongly modulated by external oscillatory drive when the drive frequency was in the cellular resonance frequency range. We observed that, with increasing strength of excitatory synaptic coupling strength w_E_, the system gradually transitions between distinct dynamical regimes (listed in order of increasing w_E_): 1) ordered, input dependent spiking resonantly driven by external oscillatory drive, 2) synchronous phase locked network firing modulated via resonant external oscillatory drive, 3) PING-like mediated gamma/theta cross frequency coupling, and 4) highly-synchronous single bursting oscillation. The first two regimes are driven and/or mediated by oscillatory resonance of neuronal subthreshold oscillations and external oscillatory drive, and occur for weak w_E_. In these regimes, even though the excitatory connectivity is weak, synchronization is dominated by excitation. Synchrony in regimes three and 4 with high w_E_ is mediated by PING-like mechanisms dominated by the periodic shunting effect of inhibition. These results indicate that the network synchronization mechanisms gradually switch from resonance regimes to PING regimes. These regimes are reflected in the total current-vs-E/I ratio trajectory curve with the second turning point defining the transition between the two synchronization regimes. The first turning point in the trajectory curve indicates the initiation of a more dominant effect of inhibition in network dynamics.

We find that in the resonance regimes (1 and 2) an external oscillatory drive near resonant frequency coupled to subthreshold voltage oscillations provides a global temporal readout mechanism for network states represented by the level of the external drive to individual cells. Specifically, within synchronous bursts the excitability of neurons receiving different magnitudes of input is mapped onto their relative spike times. These differences in spike times then result in different phases of firing relative to the external drive. In brain activity, spontaneous ordered spiking during sleep or sequential firing of place cells in hippocampus after spatial learning has been observed experimentally (Foster and Wilson 2006; Wikenheiser and Redish 2013). Moreover, a frequency dependent change in firing frequency after NREM sleep consolidation, which could potentially be a direct outcome of such a dynamical spike organization, was also observed in visual cortex (Clawson et al., 2018; Puentes-Mestril et al., 2019). Our model results predict that weak synaptic coupling with resonant activation is necessary for this firing pattern to emerge (Figure 5).

As w_E_ gets stronger, loose oscillatory firing starts to form spontaneously in the network. Here, external oscillatory drive stabilizes network synchronous oscillations by controlling inter-burst intervals. This potentially provides a dependable mechanism for temporal coding which is unaffected by fluctuations in synaptic strengths or instantaneous E/I balance (Figure 4). Generally, in this regime, the oscillatory drive recruits excitation more effectively than inhibition due to the fact that cells are hyperpolarized, leading to larger driving forces of excitatory currents.

In regimes three and four characterized by higher w_E_ values, a PING-like mechanism emerges with inhibition fully dominating the generation of network synchronization (Figure 3). The random firing within bursts turns into synchronous spiking on a much faster time scale, generating nested, phase-coupled theta and fast gamma oscillatory activity (Figure 4). In the brain, such coordinated oscillations are proposed to be essential in precise neuronal communication (Fries 2005). In particular, gamma rhythmic firing has been well studied as a facilitator of inter-regional communication (Buzsáki and Watson 2012; Akam and Kullmann 2014; Bastos et al., 2015), as fast gamma band activity may be able to carry local information that is propagated sequentially via different cycles of the global theta oscillation (Sirota et al., 2008; Tort et al., 2009) (Li et al., 2012).

With even stronger excitatory coupling, highly synchronous, seizure-like bursting appears due to extreme levels of coordinated inhibition in tight temporal correlation with excitation. Thus abnormally potentiated excitatory synapses may result in abnormal neural states, which in the neocortex is demonstrated to result from phase coherent currents (Breton et al., 2019).

These last two regimes are independent of neural resonance properties but only depend on structural network properties, coinciding with previous results observed in similar networks consisting of type 1 neurons (Wu et al., 2020).

Here, we considered generic, small, randomly connected, directed networks that represent local circuits within larger cortical or hippocampal areas. The networks contained equal numbers of E and I cells so that the relative contributions of excitatory and inhibitory signaling in the network could be directly compared and controlled by the relative values of the synaptic strengths, w_E_ and w_I_. In most brain networks, E cells outnumber I cells with a ratio of about 10:1, and we have previously shown that qualitatively similar E/I regulation occurs in networks with more physiologically accurate fractions of cell types (Wu, Aton et al., 2020). Additionally, the networks considered here had a fixed connectivity density (3%) that is within the range of reported estimates for local connectivity in hippocampal brain areas, but higher than the reported median (Tecuatl et al., 2021).

Regarding the robustness of our results to varying connectivity densities, we note that our results focus on synchronization in networks of neurons with type 2 excitability and resonance properties, that show high susceptibility for synchronization with sufficient excitatory synaptic signaling. Previous modeling work has shown that with respect to the generation of synchrony in these types of networks, connectivity density and synaptic weight can compensate for each other, particularly for excitatory density and synaptic weight (Bogaard et al., 2009; Rich et al., 2017). For the results reported here, we expect that, for networks with lower connectivity density, dynamics similar to regime 1, characterized by large effects of external resonant drive, would persist for larger ranges of w_E_ values. However, regime four dynamics, which exhibit the tightest synchrony across the entire network, may not be achieved even for the highest w_E_ values. This is because a sufficient level of inhibitory shunting (which is driven by sufficiently synchronized excitatory drive) required to generate PING-like synchrony may not be achieved. Further, the extent of w_E_ intervals displaying dynamics of regimes 2 and three would be sensitive to the w_I_ value and inhibitory connectivity density. Specifically, sufficiently high inhibitory signaling would be needed to achieve PING-like synchrony. With lower inhibitory connectivity density, we further expect that modulation of network dynamics by external resonant drive may be more dominant in all dynamical regimes.

On the other hand, for networks with higher connectivity density we expect that regime 1 dynamics would transition to regime 2 dynamics for lower w_E_/w_I_ values, as synchronization through excitatory signaling would be promoted. Likewise, regimes three and four dynamics may be achieved for lower w_E_ values, depending on the w_I_ value. Modulation of network activity by external resonant drive may be reduced in networks with higher connectivity density, as synaptic signaling would more strongly influence cell responses.

Finally, while the specific values of network parameters considered here may not be representative of all brain networks, the network dynamics we explore occur between regimes where excitatory and inhibitory signaling is equal, namely between the two crossings of the E/I trajectory loop through the point (E/I ratio = 1, total synaptic current = 0 μA). Thus, our results pertain to physiologically reasonable regimes.

Our modeling results provide insight into possible network transitions through regulation of E/I balance and oscillatory tone. It provides a basis for coexistence of multiple communication schemes in the hierarchical organization of the brain (Hahn et al., 2019). We conclude that changing E/I levels in the network mediate these diverse communication schemes. Our findings provide clear predictions for continued experimental studies of E/I balance regulation and network dynamic transitions.

## Data Availability

The original contributions presented in the study are included in the article/Supplementary Materials, further inquiries can be directed to the corresponding authors.
