# Supplementary material for: Heterogeneous mechanisms for synchronization of networks of resonant neurons under different E/I balance regimes
Source: Front Netw Physiol. 2022 Sep 30;2:975951. doi: 10.3389/fnetp.2022.975951 (PMC10013004; doi:10.3389/fnetp.2022.975951)
Supplement: Supplementary file 1 [file DataSheet1.pdf]

Heterogeneous mechanisms for synchronization of networks of resonant neurons under different E/I balance regimes

Jiaxing Wu[1], Sara J. Aton[2], Victoria Booth[3,4], Michal Zochowski[1,5,6]

1. Applied Physics Program, University of Michigan, Ann Arbor, MI 48109
2. Department of Molecular, Cellular and Developmental Biology, University of Michigan, Ann Arbor, MI 48109
3. Department of Mathematics, University of Michigan, Ann Arbor, MI 48109
4. Department of Anesthesiology, University of Michigan Medical School, Ann Arbor, MI 48109
5. Department of Physics, University of Michigan, Ann Arbor, MI 48109
6. Biophysics Program, University of Michigan, Ann Arbor, MI 48109

Supplemental Material

In this Supplemental Material we present results showing how synaptic inhibitory strength qualitatively modulates the results shown in the main text. We track the trajectories of E/I ratio and the difference between E and I currents (total current) as excitatory synaptic strength w_E is varied for three different values of inhibitory synaptic strength w_I (Fig. S1a). We compare the shape of the trajectory loops for three different values of w_I when the network is driven with external oscillatory current input in the resonant frequency range (5Hz) to when it is not driven (0Hz). We observe that increased inhibitory synaptic strength limits the maximum E/I ratio and thus the domain of the trajectory loop, which is further diminished if the resonant drive is present.

The trajectory loops for all three w_I values illustrate the two synchronization regimes discussed in the manuscript. In the resonance regime for lower w_E values, resonant oscillatory drive constrains the trajectory around E=I balance points with smaller trajectory loop sizes (dashed lines) than when no drive is given (solid lines). In the network-driven PING-like synchronization regime for higher w_E values, external oscillatory drive does not have pronounced effects.

At the same time, the trajectories with various w_I values show marked differences. First, the size of the trajectory loop in the resonance regime decreases with stronger inhibition. For w_I=3mS/cm^2, the stronger inhibition affects the activity of inhibitory cells diminishing overall inhibitory signaling, so that the network does not exhibit single spike bursting activity even at the strongest w_E range. In addition, the network does not enter the inhibition-dominant regime for highest w_E values (total current < 0 and E/I ratio <1) even though w_I is high. This indicates that the excitatory and inhibitory signals interact in a recurrent, non-linear way.

Figures S2 and S3 depict sample raster plots for higher inhibitory synaptic strength values, w_I=1mS/cm^2 and w_I=3mS/cm^2, respectively. In both cases, similarly as for w_I=0.3mS/cm^2 in the main text, when w_E is weak, the resonant oscillatory drive mediates ordered spiking within synchronous bursts (middle row, left side). For slightly higher w_E, resonant oscillatory drive increases the spiking synchrony through phase locking. The colored markers (stars and dots) denote parametric locations from Fig. S1.

However, in the PING-regime, the behavior of the networks for different inhibition w_I values diverges. For w_I=1mS/cm^2 (Fig. S2, right side), with the increase of w_E, the network firing pattern changes from wide (multi-spike) bursts to narrow, often single spike bursts. When w_E has medium values (w_E=1.5mS/cm^2, right side, first column), cells fire randomly at a high rate during network bursts rather than synchronously, due to the weakened inhibitory signal. For higher w_E (w_E=3mS/cm^2, right side, 2nd column), inhibitory cells get higher excitatory signaling and generate stronger inhibition, which causes shorter network bursts. For higher inhibitory synaptic strength (w_I=3mS/cm^2, Fig. S3, right side), however, due, to strong inhibitory-to-inhibitory connections, inhibitory signaling is not strong enough to generate single spike bursts, and network bursts are long-lasting. For both levels of inhibitory synaptic strength (w_I=1mS/cm^2 and w_I=3mS/cm^2, Figs. S2 and S3, right sides), resonant (5Hz) external oscillatory drive phase-locks the onset of network bursts.

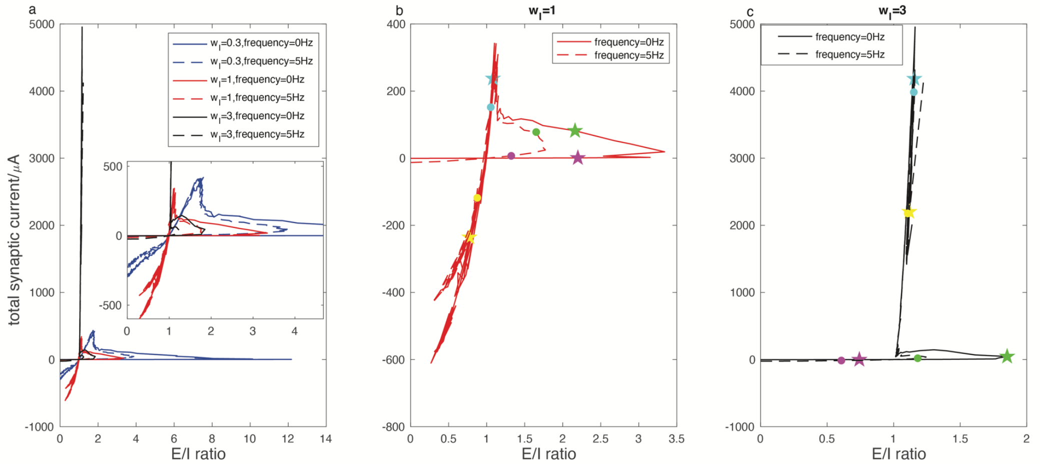

Figure S1. Trajectories of E/I ratio and E – I total current as excitatory synaptic strength w_E is increased for networks with different inhibitory synaptic strength w_I values. In panel (a), trajectories are shown for w_I=0.3mS/cm^2 (blue, same curve as Fig.1), w_I=1mS/cm^2 (red) and w_I=3mS/cm^2 (black) with no external oscillatory drive (solid lines) and with 5Hz drive (dashed lines). The details around E=I balance are displayed in the inset. The magnified curves for w_I=1mS/cm^2 and w_I=3mS/cm^2 are shown in panel (b) and (c), respectively; markers label the data points for which raster plots are displayed in Figs. S2, S3.

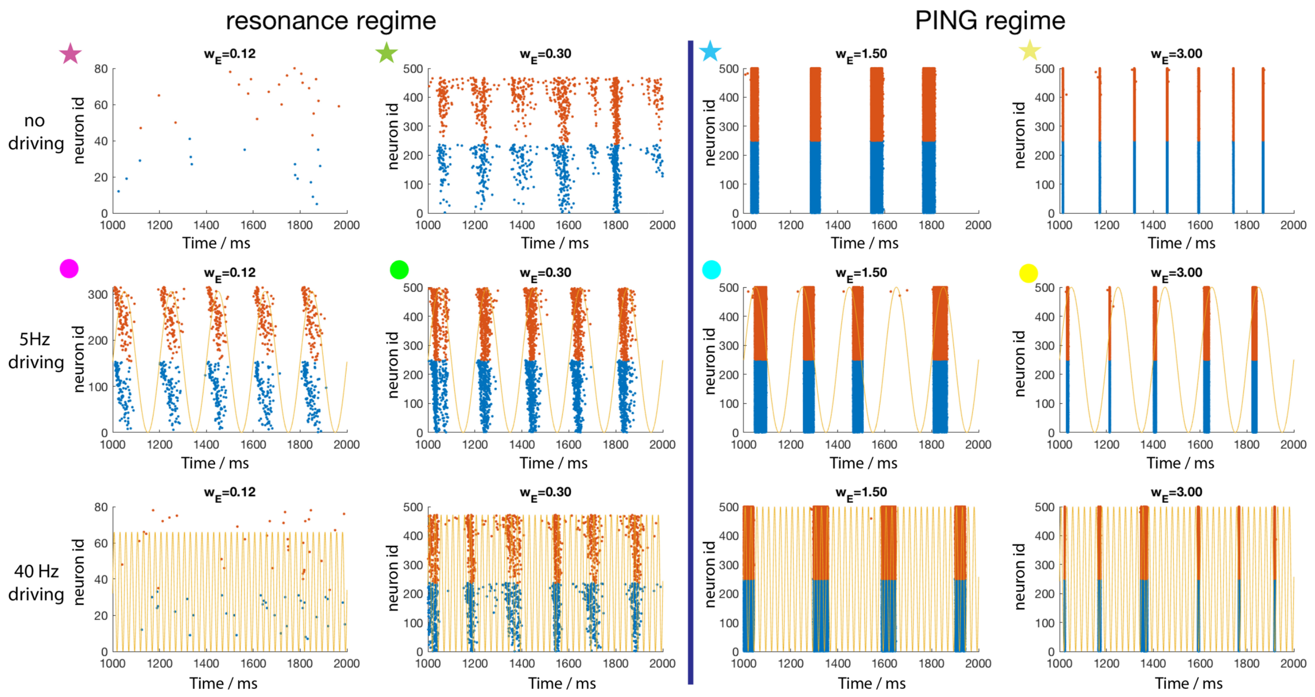

Figure S2. Example raster plots for networks with w_I=1mS/cm^2 at parameter points marked on the trajectory in Fig S1b. Top row: no external oscillatory drive; middle row: resonant (5Hz) drive; Bottom row: 40Hz (non-resonant) drive (not shown in Fig. S1b).

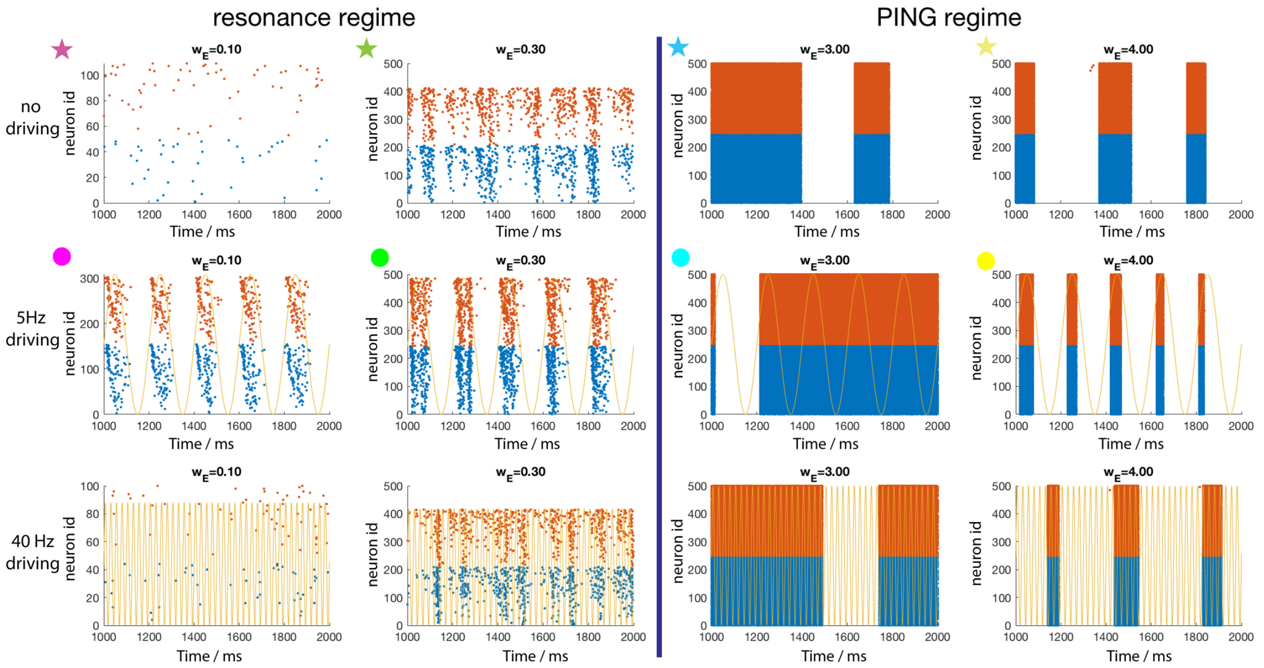

Figure S3. Example raster plots for networks with w_I=3mS/cm^2 at parameter points marked on the trajectory in Fig S1c. Top row: no external oscillatory drive; middle row: resonant (5Hz) drive; Bottom row: 40Hz (non-resonant) drive (not shown in Fig. S1b).
